# A Spinal Cord Window Chamber Model for *In Vivo* Longitudinal Multimodal Optical and Acoustic Imaging in a Murine Model

**DOI:** 10.1371/journal.pone.0058081

**Published:** 2013-03-14

**Authors:** Sarah A. Figley, Yonghong Chen, Azusa Maeda, Leigh Conroy, Jesse D. McMullen, Jason I. Silver, Shawn Stapleton, Alex Vitkin, Patricia Lindsay, Kelly Burrell, Gelareh Zadeh, Michael G. Fehlings, Ralph S. DaCosta

**Affiliations:** 1 Institute of Medical Science, University of Toronto, Toronto, Ontario, Canada; 2 Toronto Western Research Institute, Krembil Neuroscience Program, University Health Network, Toronto, Ontario, Canada; 3 Ontario Cancer Institute, University Health Network, Princess Margaret Hospital, Toronto, Ontario, Canada; 4 Department of Medical Biophysics, University of Toronto, Toronto, Ontario, Canada; 5 Department of Radiation Physics, University Health Network, Princess Margaret Hospital, Toronto, Ontario, Canada; The University of Chicago, United States of America

## Abstract

*In vivo* and direct imaging of the murine spinal cord and its vasculature using multimodal (optical and acoustic) imaging techniques could significantly advance preclinical studies of the spinal cord. Such intrinsically high resolution and complementary imaging technologies could provide a powerful means of quantitatively monitoring changes in anatomy, structure, physiology and function of the living cord over time after traumatic injury, onset of disease, or therapeutic intervention. However, longitudinal *in vivo* imaging of the intact spinal cord in rodent models has been challenging, requiring repeated surgeries to expose the cord for imaging or sacrifice of animals at various time points for *ex vivo* tissue analysis. To address these limitations, we have developed an implantable spinal cord window chamber (SCWC) device and procedures in mice for repeated multimodal intravital microscopic imaging of the cord and its vasculature *in situ*. We present methodology for using our SCWC to achieve spatially co-registered optical-acoustic imaging performed serially for up to four weeks, without damaging the cord or induction of locomotor deficits in implanted animals. To demonstrate the feasibility, we used the SCWC model to study the response of the normal spinal cord vasculature to ionizing radiation over time using white light and fluorescence microscopy combined with optical coherence tomography (OCT) *in vivo*. *In vivo* power Doppler ultrasound and photoacoustics were used to directly visualize the cord and vascular structures and to measure hemoglobin oxygen saturation through the complete spinal cord, respectively. The model was also used for intravital imaging of spinal micrometastases resulting from primary brain tumor using fluorescence and bioluminescence imaging. Our SCWC model overcomes previous *in vivo* imaging challenges, and our data provide evidence of the broader utility of hybridized optical-acoustic imaging methods for obtaining multiparametric and rich imaging data sets, including over extended periods, for preclinical *in vivo* spinal cord research.

## Introduction

Most *in vivo* imaging of the spinal cord in animals (and humans) has been conducted using computed tomography (CT), magnetic resonance imaging (MRI), diffusion tensor imaging (DTI) or ultrasound imaging [1], [2], [3], [4]. While these non-invasive imaging techniques allow *in vivo* serial imaging of the cord in preclinical models, image resolution is suboptimal for visualizing vital microscopic anatomical structures, such as the vasculature and neural tracts. Furthermore, such imaging techniques suffer from poor tissue specificity, and typically require an exogenous contrast agent to differentiate vasculature from solid tissue structures. Alternatively, optical imaging could provide a unique and powerful method of studying the intact spinal cord and its vasculature *in situ* at structural and functional levels longitudinally and at sub-micrometer resolutions (e.g. at the cellular level). However, the anatomy and location of the intact spinal cord is close to the heart and lungs, and therefore results in cord motion during imaging. Thus, *in vivo* spinal cord imaging contains inherent challenges for optical imaging compared to other central nervous system (CNS) targets, such as the retina or cerebral cortex, which can be readily accessed using *in vivo* optically-based imaging techniques, either directly or via intracranial transparent window chamber implants, respectively [1], [5], [6], [7]. Moreover, the vascular structures of the spinal cord are predominantly located in the grey matter, making it difficult to image using traditional microscopy techniques, such as confocal fluorescence microscopy as they are unable to penetrate deep enough into the spinal cord tissue to image the microvasculature of the grey matter [8], [9].

To date, a few published reports have emerged on the use of optical microscopy to visualize the mouse spinal cord *in vivo*. For example, Kerschensteiner *et al.* used *in vivo* fluorescence imaging to monitor individual fluorescent axons in the spinal cords of living transgenic mice over several days after spinal injury [10]. Davalos *et al.* used two-photon fluorescence imaging to study multiple axons, microglia and blood vessels in the mouse spinal cord *in vivo* [11]. Johannssen *et al.* labeled the superficial dorsal horn populations with a Ca(2+) indicator, and were able to stabilize the spinal cord sufficiently to permit functional imaging in anaesthetized mice using two-photon fluorescence Ca(2+) microscopy [12]. Again, using two-photon fluorescence microscopy, Kim *et al.* studied the migration of GFP(+) immune cells in the spinal cord of CXCR6(gfp/+) mice during active experimental autoimmune encephalomyelitis using an intervertebral window approach [13]. Dray *et al.* have successfully followed the dynamics of degeneration-regeneration of injured spinal cord axons while simultaneously monitoring the fate of the vascular network in the same animal up to 4 months post-injury using multiphoton fluorescence microscopy [14]. Finally, Codotte *et al.* recently demonstrated the use of optical coherence tomography (OCT) for structural and vascular imaging of the mouse spinal cord without the use of a contrast agent; however, their studies did not include repeated *in vivo* imaging [15]. These examples reflect a major recent trend in spinal cord research to apply established optical microscopy techniques to study the cord and its vascular network *in situ* and over time at high resolution and *in vivo*. However, a major drawback of all these approaches has been the need for repeated surgeries to the vertebral column of the same animal to expose the cord or, alternatively, animal sacrifice for *ex vivo* tissue analysis.

**Figure 1:**
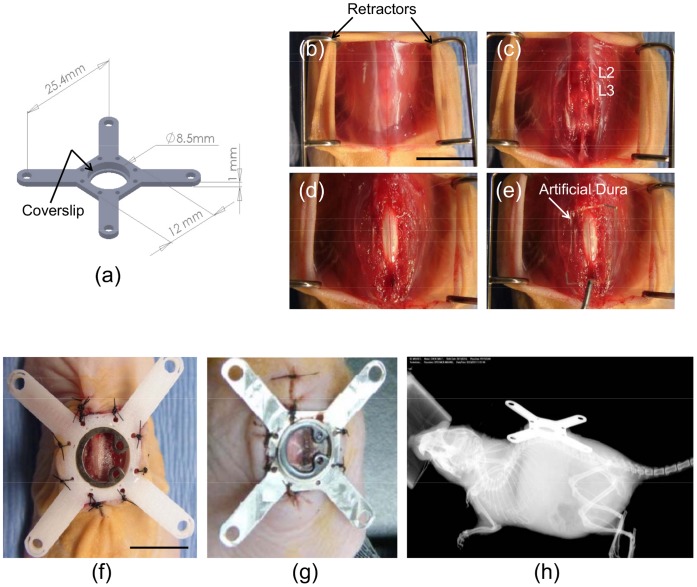
Mouse spinal cord window chamber (SCWC) device and surgical implantation procedures. (A) The SCWC device design and dimensions are shown. An 8 mm diameter glass coverslip was inserted into the SCWC device and held in place by a metal ring clamp once the device is surgically implanted into the mouse (shown in panel “F” and “G”). Four radial extension arms have been built into the device in order to immobilize the animal during imaging sessions. (B-E) Photographs showing *step-by-step* surgical procedures for implanting the SCWC in the mouse exposing the spinal cord at the L2–L3 vertebrae. (E) Artificial dura was placed on the dorsal surface of exposed spinal cord below the coverslip to prevent scar tissue formation. Two separate devices were manufactured from either durable (F) polycarbonate or (G) light-weight surgical steel. Polycarbonate was used to allow photoacoustic imaging *in vivo*. (H) X-ray images were taken following SCWC implantation to confirm the device had been placed over L2–L3 and demonstrate that the spinal cord and vertebrae remain structurally sound after implantation of the device. Scale bars = 1 cm.

Recently, Farrar *et al.* reported that they had overcome the limitation of repeated surgical procedures by using a metal spinal cord window chamber implanted between T11–T12 of the mouse vertebral column for repeated optical imaging [16]. Briefly, the spinal chamber held a glass coverslip in place and provided continuous optical access to the cord for over five weeks, allowing quantitative imaging of microglia and afferent axon dynamics after laser-induced damage to the cord. Fenrich *et al*. also recently developed a SCWC model to examine axonal regeneration following a ‘pin-prick’ model of spinal cord injury [17]. While these studies provide elegant designs for longitudinal *in vivo* spinal imaging, both models utilize metallic components and conduct multiphoton microscopy for high-resolution image acquisition. However, metal devices are incompatible with other emerging optically-enabled imaging techniques which could provide additional complementary biological information about the cord and, in particular, its vasculature. For example, photoacoustic imaging [18], which combines optical excitation and ultrasound detection, can provide quantitative information about the vasculature throughout the the full thickness of the cord at imaging depths unacheivable with mutliphoton fluorescence microscopy. In addition, multispectral photoacoustics can provide quantitative information about the oxygenation status of the cord vasculature [19], [20]. Power Doppler ultrasound can also be used to determine vascular density *in vivo*. Thus, while the window chamber approach of Farrar *et al.* is a significant step forward for *in vivo* optical imaging of the mouse spinal cord, it is limited to mutliphoton fluorescence microscopy. Here, we report the development and testing of an alternate design of a transparent spinal cord window chamber (SCWC) implantable device composed of either metal or polycarbonate materials for mice (Figure 1; and rats, *See File S1 and Figure S1*) that overcomes the need for repeated spinal surgeries. We demonstrate the feasibility and utility of our approach to obtain multiparametic (morphological, structural, functional, and cellular) high-resolution imaging data of the mouse spinal cord and its vasculature using multiple complementary imaging techniques (including fluoresence microscopy, OCT, power Doppler ultrasound and photoacoustic imaging) longitudinally and *in vivo*.

## Methods

All animal procedures were conducted with approval from the University Health Network Animal Care Committee (Animal Use Protocol #2263 and #2609).

### Mouse Window Chamber Designs

The spinal cord window chamber (SCWC) devices were modeled and designed using SolidWorks® software (SolidWorks Corporation, Waltham, MA, USA) (Figure 1A). Window chambers were 3D printed using a Fortus 3D Production printer systems (Stratasys, Eden Prairie, MN, USA) using ABS-polycarbonate (Figure 1F) or machined to the same specifications out of surgical grade stainless steel (Figure 1G). The metal SCWC devices were used for X-ray irradiation experiments and subsequent fluorescence and speckle-variance OCT (svOCT) imaging. Since the metal device was thinner, it allowed for closer contact between the tissue and imaging objective lenses. However, since metal is incompatible with photoacoustic imaging, polycarbonate SCWC devices were installed in animals for the photoacoustic and power Doppler ultrasound imaging. Both metal and polycarbonate SCWC designs had eight circumferentially-located holes for surgical sutures to secure the device to the dorsal skin. The total weight of SCWC devices were 0.35 g (plastic) and 1.0 g (metal), which were well tolerated by the mice. The SCWC coverslip had a diameter of 8 mm, permitting use of water-coupled high-magnification microscope objective lenses for high-resolution imaging *in vivo*. To restrain the mouse to the microscope stage during imaging, four perpendicular extension arms were added to the device to mechanically screw the animal to the microscope stage, thus ensuring stability and minimizing movement during intravital imaging (Figure 1). Standard glass coverslips of 8 mm diameter (Cat. No. 5DE89, Grainger, Lake Forest, IL, USA) was used for the mouse SCWC. Coverslips were held in place by a metal ring clamp (Cat. No. 5DE89, Grainger, Lake Forest, IL, USA) once the device was implanted in the animal.

### Mouse Spinal Cord Window Chamber Installation

Female athymic nude mice (NCRNU-F, Taconic, Hudson, NY, USA) or C57BL6 (Jackson Laboratories, Bar Harbor, Maine, USA) at 15–20 weeks, were anesthetized using a mixture of ketamine (80 mg/kg) and xylazine (5 mg/kg) prior to surgical installation of the SCWCs. Briefly, mice were placed in a sterile surgical preparation area and the dorsal skin was disinfected with 70% isopropyl ethanol and 10% povidone-iodine. A 3–4 cm incision, using a #15 blade scalpel, along the dorsal midline was made in the lumbar region to expose the spine (Figure 1B), and a two-level laminectomy at L2–L3 was performed using fine scissors (Figure 1C and 1D). After exposing the spinal cord, India ink (Pelikan, #221143, Hannover, Germany) was carefully applied to the middle of the spinal cord using the tip of a sterile piece of tissue paper, approximately the size of a 30 gauge needle. This served as a landmark for longitudinal imaging, enabling us to locate and track the same landmark between multiple imaging sessions. Then, a small piece of customized artificial dura, made of thin pliable and biocompatible silicon rubber (Eagerpolymers, #0812, Chicago, IL, USA), was placed over the spinal cord to prevent scar tissue formation (Figure 1E). The artificial dura was prepared by polymerizing the optically transparent silicone rubber with a curing agent (Cat. #0812, Eagerpolymers, Chicago, IL, USA) to form a thin membrane which was spread in a Petri dish to a thickness of 0.25 mm. A similar application of silicon rubber was used by Shtoyerman *et al.* [21]. When the biocompatible artificial dura was fully polymerized after 12 h, it was custom cut to size in order to cover the exposed area of the spinal cord.

A sterile, light-weight SCWC device (Figure 1A) was implanted and fixed to the superficial dorsal muscles and skin using standard nylon sutures (Covidien, Syneture Monosof 5-0 sutures, Norwalk, CT, USA) (Figure 1B). The SCWC was sutured tightly inside the incision, with any additional skin being sutured together to create a seal around the device. An 8 mm diameter glass coverslip was placed inside the inner ring of the mounting device and then held in place using a thin 8 mm diameter metal retaining ring. Animals were administered anesthetics and underwent surgical procedures for approximately 30 minutes each. Following surgical implantation of the device, mice were transferred to a temperature-controlled recovery pad until awake and then returned to their cages to fully recover. Animals were given oral antibiotics (Clavamox®) in water for 3 days following SCWC implantation to prevent infection. At all times, with the exception of imaging sessions, animals were allowed free access to food and water. Animals were monitored daily by veterinary staff for adverse effects of the SCWC implants and any signs of decreased mobility, infection, necrosis, or window chamber dehiscence.

### Histology and Immunostaining

**Figure 2:**
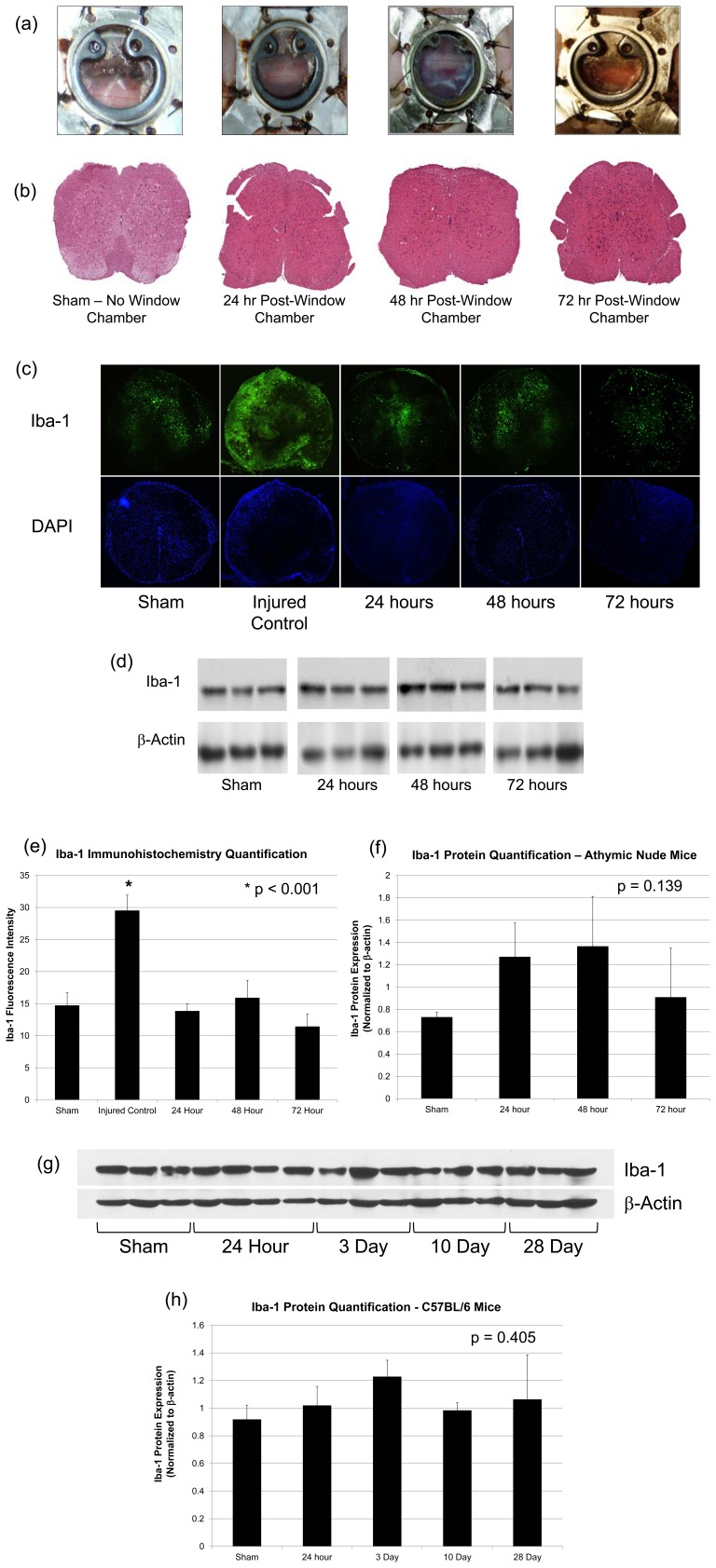
Visual and histological confirmation that SCWC implantation does not damage the spinal cord structure or cause significant inflammation or infection. (A) White light images following SCWC implantation at 0, 1, 3 and 7 days showed no signs of local infection, excessive bleeding around the installation site, or device rejection. SCWC remained optically clear for 29 days, permitting long-term high-resolution imaging of cord and vascular structures. Yellow arrows indicate the location of the spinal cord. (B–E) Histological analysis and quantification of spinal cord tissue cross-sections cut directly below the caudal edge of the implanted SCWC. (B) H&E staining confirmed tissue morphology was intact after WC implantation. (C) Representative Iba-1 immunohistochemistry images from spinal cords 24, 48 and 72 hours following SCWC implantation. Sham and spinal cord injury (SCI) (Iba-1 positive control) animals are also shown for comparison. SCI positive control animals showed a significant increase in Iba-1 expression, * p<0.001. No notable changes in Iba-1 expression in *ex vivo* spinal cord were observed between the SCWC implanted groups). (D) Western blot for Iba-1 prior to SCWC implant (sham), and at 24, 48, and 72 hours post-implantation in athymic nude mice. (E) Bar graph representing the fluorescent intensity quantification of Iba-1, and no significant increase in Iba-1 was observed; n = 3 per group (p-values >0.212 for comparisons between all groups). (F) Bar graph showing quantification of Western blot data. Increases in Iba-1 protein were observed in animals receiving SCWC implantation, although these increases were insignificant (p = 0.15); n = 3 per group. (G) Western blot for Iba-1 prior to SCWC implant (sham) (n = 3), and at 24 hours (n = 4), 3 days (n = 3), 10 days (n = 3) and 28 days (n = 3) post-implantation in C57BL6 mice. (H) Bar graph showing quantification of Western blot Iba-1 data (C57BL6 mice). No significant increases in Iba-1 protein were observed (p = 0.405). It is anticipated that the slight increases in Iba-1 in the athymic nude mice may be due to the laminectomy and surgical procedures performed. β-Actin was used as a protein loading control. Scale bars = 400 µm. Data was transformed (square-root transformation) and analyzed using a one-way ANOVA; Tukey post-hoc analysis. SCWC = spinal cord window chamber.

To investigate the presence of an inflammatory or immune response in the spinal cord caused by the surgical implantation of the metallic and plastic SCWC devices, mice bearing chambers were deeply anesthetized and transcardially perfused with 10% formalin at 24, 48 and 72 h after implantation (n = 3 per group; total n = 9) (Figure 2). Sham animals (naïve, no surgery or SCWC implantation) were used as a control (n = 3). A 5-mm long section of the spinal cord from directly below the SCWC was extracted and fixed in 10% formalin for 24 hours and then embedded in paraffin for tissue sectioning and histological staining. Tissues were cut in longitudinal and axial sections, serially at a thickness of 4 µm, and fixed onto glass microscope slides (VWR, #48311-703, Canada). Sections were stained with hematoxylin and eosin to determine anatomical and cellular microstructures, and with Iba-1 (1∶300, Cat. # 019-19741, Wako Chemicals USA, Richmond, VA, USA) to assess inflammation following SCWC device implantation [23], [24]. Positive controls were used to confirm Iba-1 reactivity of the antibody (mouse spinal cord tissue from 7 days post-injury was used from animals receiving an 8 g clip-compression spinal cord injury, as described previously by Yu and Fehlings [25]).

White light micrographs were obtained of the H&E stained tissue sections using a stereoscopic epifluorescence microscope (Leica MZ FLIII, Leica Microsystems, Richmond Hill, ON, Canada). For Iba-1, tiled images were taken at 20X magnification using StereoInvestigator® software (MicroBrightField Inc., Williston, VT, USA), and images were quantified for fluorescent intensity using ImageJ software (National Institutes of Health). Data from each group was subject to a square-root transformation (to adjust for any uneven distribution of normality and/or variance within groups), and then a one-way ANOVA, with a Tukey post-hoc analysis, was used to analyze the Iba-1 fluorescent intensity data between control animals and SCWC animals at various time points following implantation.

### Western Blot Analysis

Following deep sedation, animals were sacrificed by decapitation at 24, 48 or 72 hours following SCWC implantation (n = 3 per group; total n = 9). Sham animals (naïve, no surgery or SCWC implantation) were used as a control (n = 3). A 10 mm length of the spinal cord centered under the SCWC was surgically removed. Samples were mechanically homogenized in 100 µl of homogenization buffer (0.1 M Tris, 0.5 M EDTA, 0.1% SDS, 1 M DTT solution, 100 mM PMSF, 1.7 mg/mL aprotinin, 1 mM pepstatin, and 10 mM leupeptin) and centrifuged at 15,000 rpm for 10 minutes at 4°C. Supernatants were extracted and used for Western blot analysis, where 10 µg of protein was loaded into 12% polyacrylamide gels (Bio-Rad, Mississauga, Canada). Membranes were probed with primary anti-Iba-1 antibody (1∶500, Cat. # 016-20001, Wako Chemicals USA, Richmond, VA, USA). Primary antibodies were labeled with horseradish peroxidase-conjugated secondary antibodies (goat anti-rabbit IgG, 1∶2000; Jackson ImmunoResearch Laboratories, West Grove, PA, USA), and bands were imaged using an enhanced chemiluminescence (ECL) detection system (Perkin Elmer, Woodbridge, Canada). Mouse monoclonal beta-actin (Chemicon International, Inc., Temecula, CA, USA) was immunoblotted as a loading control as per standard protocol. Gel-Pro Analyzer® software (Media Cybernetics, Bethesda, MD, USA) was used for integrated optical density (OD) analysis and quantification of Iba-1 protein expression (Figure 2). Data from each group was subject to a square-root transformation (to adjust for any uneven distribution of normality and/or variance within groups), and then a one-way ANOVA was used to statistically analyze the data between naïve and SCWC implanted groups. A Tukey post-hoc was applied.

### X-ray Micro-irradiation and Vascular Injury

**Figure 3:**
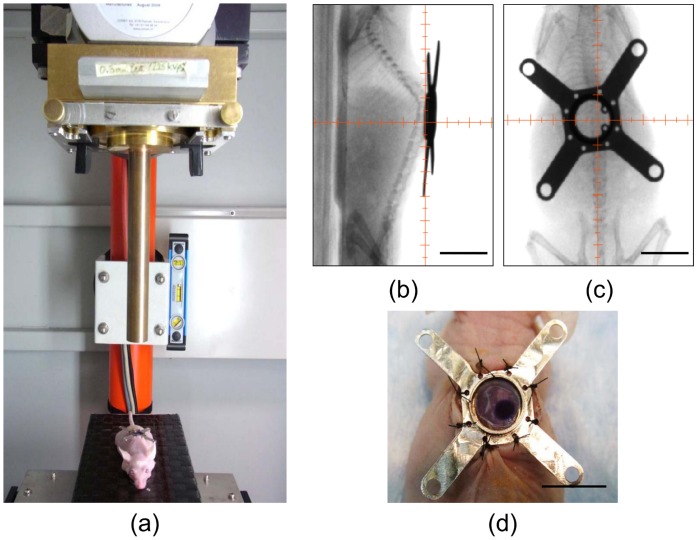
SCWC model permits X-ray microirradiation of the spinal cord *in situ*. (A) Anaesthetized mice were placed directly under the micro-irradiation collimator of the small animal irradiator for delivery of X-rays to the spinal cord through the coverglass of the window chamber. (B, C) *In situ* fluoroscopic imaging was used for image-guided delivery of the X-ray beam (centered on the crosshairs) to the spinal cord. (D) Custom fit radiochromic film was used to confirm the location of the irradiation beam which had a 3 mm diameter (as seen by the dark blue circle). Scale bar = 1 cm. SCWC = spinal cord window chamber.

To demonstrate the feasibility of using the mouse SCWC for imaging radiation response of the spinal cord and its vasculature *in vivo*, we delivered ionizing radiation to the spinal cord using a custom-designed small animal X-ray microirradiator system (XRad225Cx, Precision X-Ray Inc., North Branford, CT, USA) (Figure 3A). The fully automated microirradiator system was controlled using a computer system that integrates cone beam computer tomography (CT) imaging with focused X-ray delivery technology, and was able to deliver a single focal radiation beam at a dose of 30 Gy with a diameter of 3 mm directly to the spinal cord at 2.5 Gy/min. The X-ray tube was mounted on a rotating gantry with a flat panel detector located opposite to the isocenter, which facilitated imaging and irradiation of the target at any given angle. The irradiator was calibrated to ensure accurate dose delivery with tissue phantoms using methods previously described [22].

Prior to irradiation, mice were anaesthetized by intraperitoneal injection of ketamine (80 mg/kg) and xylazine (5 mg/kg) and were secured on the stage at the radiation isocenter (n = 5). Fluoroscopy images of anatomical features of the animal and the integrated targeting software were used to align the center of the target to the isocenter of the radiation beam in the three axes (X,Y,Z) by automatic movement of the stage for an anterior-posterior (AP/PA) radiation treatment. Radiation dosimetry was performed using radiochromic EBT film (ISP Inc., Wayne, NJ, USA) consisting of a radiosensitive monomer that polymerizes upon irradiation. A white light image of the mouse was taken using a stereoscopic epifluorescence microscope (Leica MZ FLIII, Leica Microsystems, Richmond Hill, ON, Canada) immediately after X-ray irradiation in order to visualize and spatially define the radiation field to permit accurate spatial localization of the treatment dose for subsequent intravital fluorescence imaging.

### Intravital White Light and Fluorescence Imaging

*In vivo* white light and fluorescence imaging were performed on mouse spinal cords at 1, 24, 48 hours, and up to 5 days after irradiation (n = 5). Prior to each imaging session, mice were anaesthetized by intraperitoneal injection of ketamine (80 mg/kg) and xylazine (5 mg/kg) and placed within the custom-made animal restraint and secured to the microscope stage with an embedded heating pad to maintain the animal’s body temperature during imaging.

White light and fluorescence macroscopic images of the spinal cord were acquired through the transparent glass coverslip of the window chamber using a stereoscopic epifluorescence microscope (Leica MZ FLIII, Leica Microsystems, Richmond Hill, ON, Canada). To visualize the spinal cord vasculature, FITC-conjugated dextran (0.65 mg/mouse, MW = 20 kDa; Sigma–Aldrich Corporation Ltd, Oakville, Canada) was administered intravenously by tail vein prior to fluorescence imaging, and then imaged using a 470 nm excitation filter set. Using this method, macroscopic imaging allowed for the determination of radiobiological changes to the spinal cord tissue and vasculature at the sub-millimeter scale.

### Intravital Bioluminescence and Fluorescence Imaging of Tumor Metastases In Vivo

To demonstrate the feasibility of using the mouse SCWC for imaging tumor micrometastases *in vivo*, we used an inverted confocal fluorescence imaging microscope (LSM510 Laser Scanning Confocal Microscope, Carl Zeiss, Jena, Germany) to visualize microvasculature and the micrometastases of WW426 medulloblastoma cells (both fluorescent and bioluminescent, expressing a c-myc-GFP tag and Luc-RFP reporter construct) which were injected intracranially 28 days prior to *in vivo* imaging. *Cell culture:* WW426 cells were grown as adherent culture using DMEM:FBS(10%) heat inactivated in standard non-treated tissue culture. The cell line is predisposed for myc-C(GFP) cells to become unattached, but the GFP (myc) signal has a positive feedback loop whereby high myc-C(GFP) signal is required to sustain high levels of myc-C(GFP). Thus, it was essential that the floating cells are retained in the culture both during feeding and splitting. When the cells reached 60–80% confluency, they were split 1∶3 (approximately every 5 days). Old media was removed from the flask, with floating cells, and kept in a 15 mL tube. 1 mL trypsin was added to the flask and then placed at 37°C for 5 minutes, or until the cells dissociated. The trypsin was neutralized with existing culture media, and the suspension was centrifuged to retrieve all cells.

#### Cell transplantation

The skulls of athymic nude mice (n = 10) were surgically exposed and bur-holes were carefully drilled to allow access to the posterior cerebellum. 4×10^5^ cells (total volume 10 µl) were injected 2–3 mm deep into the cerebellum at a rate of 2 µl/minute (n = 3). The needle remained in the brain for 1 minute after the injection to prevent fluid backflow.

The primary intracranial WW426 medulloblastoma tumors took approx. 3 weeks to grow and then metastasize to the spinal cord, and some animals did not develop spinal metastases in these experiments. Our intention was to determine whether our WC model was capable of imaging tumor micrometastases occurring in the spinal cord *in vivo*, as a proof-of-concept. To test this, we used *in vivo* bioluminescence imaging to non-invasively track the tumor growth in the brain in the whole animal from the time of the initial tumor cell implant prior to surgical implantation of the WC device, since our previous experience with this tumor line showed it was slow growing. This enabled us to implant the WC only once the primary brain tumor was sufficiently grown and metastases to the spine were likely. Bioluminescence imaging was performed using an IVIS Spectrum imaging system (Caliper, MA, USA) by injecting luciferin substrate intraperitoneally (150 mg/kg) prior to each bioluminescence imaging session. We then implanted the SCWC devices 27 days after intracranial tumor seeding and used bioluminescence to confirm the presence of medulloblastoma micrometastases within the spinal cord through the transparent coverslip window.

Since the tumor cells were GFP-positive, TRITC-conjugated dextran was used to image the vasculature, and was administered intravenously via the tail vein prior to fluorescence microscopy (100 mg/kg body weight, MW = 155,000 Da; Sigma–Aldrich Corporation Ltd, #T1287, Oakville, Canada). A Zeiss LSM510 confocal fluorescence microscope (Carl Zeiss, Jena, Germany) was used to observe the location of the micrometastases in relation to the vasculature. A 5× Fluar objective (Carl Zeiss, Jena, Germany) was used for intravital confocal fluorescence microscopy of the cord as it had a 12.5 mm (NA 0.25) working distance and allowed a wide area of the cord to be imaged without the need for tiling of multiple images. Animals were imaged 1 day following SCWC implantation (28 days post-cell transplantation).

### Optical Coherence Tomography (OCT) Imaging

Optical coherence tomography (OCT) was used for depth resolved three dimensional structural and functional imaging of the spinal cord and its vasculature *in vivo*. Imaging was performed on anesthetized mice with a swept-source OCT system described previously [22]. Briefly, a 36-kHz swept laser source with a sweeping range of 110 nm centered at 1310 nm was used to acquire depth resolved structural images of the intact *in vivo* spinal cord up to ∼2 mm in depth with an axial resolution of ∼8 µm and a lateral resolution of ∼13 µm. Three dimensional structural OCT images were acquired over 2.5 mm×3 mm regions of the cord within the window chamber.

Speckle variance OCT (svOCT) is a functional extension of OCT that enabled depth resolved three-dimensional imaging of *in vivo* spinal cord vasculature as small as ∼20 µm in diameter without the use of exogenous contrast agents [22]. The difference in the temporal speckle statistics of blood and solid tissues provides the contrast in svOCT. Three dimensional vascular images were acquired over the same 2.5 mm×3 mm region as the structural images and vascular contrast was obtained by computing the interframe speckle variance over four consecutive B-mode images. svOCT is highly sensitive to motion such as breathing and heartbeat; therefore mice were secured in a custom holding frame to minimize motion artifacts.

Unavoidable motion artifacts caused by breathing created bright streaks through the images in the scanning direction and were minimized by applying a 3×3 median filter in the depth direction, followed by a clamp to remove low-intensity pixel values.

Doppler OCT imaging enabled real-time visualization of blood flow in the posterior spinal vein. Two-dimensional B-mode Doppler images were formed using the Kasai estimator to determine the phase shift of scattering red blood cells over consecutive A-scans [23]. Doppler imaging was performed with 2000 A-scans over a 1 mm region centered on the posterior spinal vein with an ensemble length of eight. The imaging head was angled ∼15° relative to the surface of the chamber, providing a Doppler angle of ∼75°. (n = 4, for svOCT and Doppler OCT imaging. Imaging parameters were optimized during OCT sessions, using 3 mice).

### Ultrasound and Photoacoustic Imaging

*In vivo* ultrasound, power Doppler, and photoacoustic imaging of the polycarbonate (plastic – Figure 1F) SCWC-bearing animals were performed using the Vevo2100 and Vevo LAZR systems (VisualSonics Inc., Toronto, ON, Canada) with a 40 MHz centre frequency transducer (LZ-550, VisualSonics Inc., Toronto, ON Canada) at 24 hours following SCWC implantation (n = 2). These experiments were conducted as terminal, end-point procedures. The vascular hemoglobin oxygen saturation (sO_2_) was determined by irradiating the window chamber with light of two different wavelengths (750 nm and 850 nm). The built-in software on the Vevo LAZR system automatically calculated sO_2_ based on the received photoacoustic signals. The energy density of the laser beam at the surface of the window chamber was approximately 3 mJ/cm^2^_._ The glass coverslip was not acoustically compatible; therefore it was removed for these experiments. Sterile coupling gel (LithoClear, Sonotech, Washington, USA) was applied to the artificial dura above the spinal cord to facilitate the transmission of acoustic waves between the tissue and ultrasound transducer, therefore reducing air-tissue interface-based imaging artifacts. Three-dimensional co-registered power Doppler and sO_2_ measurements of the spinal cord were performed while the mouse was breathing 100% oxygen mixed with 2% isoflurane for approximately 20 minutes. In addition, during photoacoustic imaging, the hemoglobin oxygen saturation recovery dynamics of the spinal cord were measured by shifting the animal’s anesthetic mixture from 100% to 7% oxygen for 1 minute. Quantification of the sO_2_ recovery measurement was performed in a region of interest around the spinal cord in a single imaging plane. Post-processing of sO_2_ and power Doppler images was performed using Amira (Visage Imaging, San Diego, CA, USA). A median filter was applied to the sO_2_ data set to reduce the effects of clutter. The data sets were overlaid with an anatomical B-Mode image.

## Results

### Spinal Cord Window Chamber Design and Implantation

In the present study, we designed and developed two types of spinal cord window chamber (SCWC) devices (metal and plastic) and the procedures to surgically implant them in mice to permit longitudinal high-resolution multimodal optical and acoustic imaging of the spinal cord and its vasculature (Figure 1A). Our SCWC device was easily implanted following a two-level laminectomy at L2–L3 (Figures 1B–1E, 1H) with both polycarbonate (Figure 1F) and metal (Figure 1G) compositions to permit *in situ* imaging of the cord and its vasculature using a several complementary intravital optical imaging modalities. The devices were light weight and the animals tolerated them well for up to 1 month (*See Video S1*).

In a cohort of (non-irradiated) animals, we investigated the possibility of the implanted SCWC devices causing local swelling and/or infection at the surgical site, and examined the spinal tissue directly below the window chamber for tissue damage and inflammation. White light images following SCWC implantation showed no visible hallmark signs of local infection or device rejection at day 0, 1, 3 or 7 after SCWC implantation (Figure 2A). There was no microstructural damage to the cord as determined by *ex vivo* histological assessment using hematoxylin and eosin staining in animals 24, 48 and 72 hours post-SCWC implantation (Figure 2B). Furthermore, using *ex vivo* immunostaining of Iba-1, an indicator of macrophage/microglia activation and inflammation, we confirmed that there was negligible inflammation in spinal cord tissues from the time of the device implantation and up to 72 h after implantation, thereby indicating that the SCWC device did not cause injury to the cord (Figure 2C, E). In contrast, spinal cord injured (SCI) mice at 7 days post-injury, used as a positive control for Iba-1 staining, showed an increase in Iba-1 expression, which is consistent with previous reports [26], [27]. In addition, Western blot analysis and quantification of Iba-1 protein further indicated a lack of an inflammatory response in the area below the SCWC (Figure 2D, F). Although we observed a slight increase in Iba-1 protein in animals with SCWC implanted, this increase is likely due to the surgery and two-level laminectomy performed in these animals, rather than the installation of the window chamber mount itself. Sham animals, which did not receive a laminectomy, were used as the control group for Iba-1 protein quantification. Sham animals showed reduced Iba-1 expression; however, when compared to C57BL6 or athymic nude animals with SCWCs installed, no considerable changes in Iba-1 expression were observed (p = 0.15). Overall, the data suggest that implanting our SCWC design over the spinal cord is feasible *in vivo*, and does not result in any discernible damage to the spinal cord tissue.

The SCWC remained optically clear for up to 29 days of imaging, after which point the devices detached (suture failure) and tissue growth into the window chamber area prevented further imaging. On average, SWCWs remained optically clear for 21 days without need for intervention; however, if required, mild tissue growth into the window chamber area was easily removed prior to imaging, allowing imaging to be conducted out to 29 days. The replacement of coverslips between imaging sessions was simple and rapid (e.g. a few minutes). The devices that we developed were easily sterilized by autoclave (for metal device) or surgical disinfectant (for plastic device) and were compatible with commercially-available glass coverslips of standard diameter.

Based on our qualitative observations and quantitative (*ex vivo*) assessments following SCWC implantation, we observed that the SCWC devices did not cause physical or biological damage to the spinal cord or its vasculature. Thus, the surgical implantation procedure or the prolonged use of an *in vivo* SCWC did not compromise the integrity or the interpretation of imaging data obtained in order to study the effect of a given treatment by differentiating it from background biological response (e.g. that might have occurred due to inflammation after surgical implantation) (Figure 2).

### Intravital Imaging of Radiation-induced Changes to Spinal Cord Vasculature

To demonstrate the utility of the animal model, we used our SCWC model to study the biological response of the spinal cord and the vasculature to X-ray irradiation. We specifically selected a microirradiation approach to induce vascular damage, because it could be delivered in a controlled, spatially-localized, and reproducible manner using the small animal X-ray microirradiator (Figure 3A, B, C). A benefit of using an implanted SCWC device was that imaging of the cord could be performed *in vivo* before and serially after irradiation in the same animal. Thus, each animal could act as its own experimental pre-treatment control. This reduced the number of animals required for experiments, as well as controlled for individual differences in vascular organization and branching within each mouse spinal cord.

**Figure 4:**
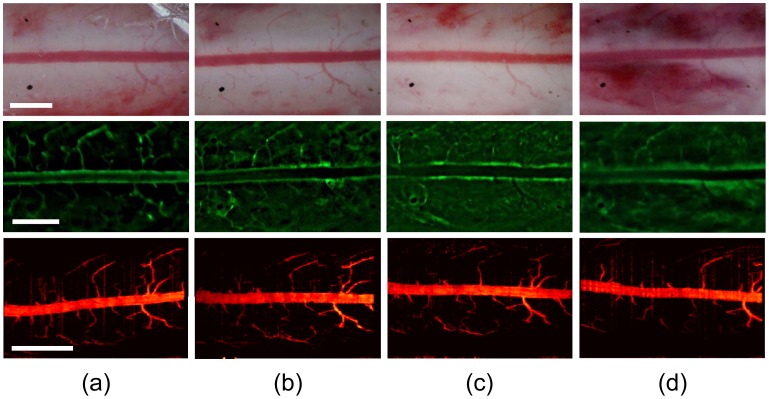
Longitudinal optical imaging of radiation response of the normal spinal cord and its vasculature through the SCWC. White light, wide-field fluorescence, and svOCT images were taken at (A) 1 day before, (B) 1 hour, (C) 24 hours, and (D) 48 hours following a single 30 Gy radiation dose to the cord. White light images revealed significant radiation-induced hematoma in the spinal cord two days after irradiation (arrows). FITC-dextran was injected intravenously prior to acquiring the fluorescence images at each time point. Fiducial markers consisting of India ink (black dots shown by arrows) on the white light images were used to as spatial landmarks to allow identification and long-term imaging of vascular structures. Compared with vascular function, corresponding en-face projected svOCT images revealed that the posterior spinal cord vein and other vasculature did not suffer significant short-term radiation-induced structural damage. Scale bar = 1 mm. SCWC = spinal cord window chamber. svOCT = speckle variance optical coherence tomography.

Consistent with previous studies of spinal cord irradiation [28], [29], [30], we observed significant radiation-induced hematoma in the spinal cord white matter two days after a single 30 Gy irradiation with a 3 mm beam diameter (Figure 3D, 4). FITC-dextran was injected intravenously prior to acquiring the fluorescence images at each time point to visualize spinal cord vasculature, and revealed significant decrease in vascular function in the posterior spinal cord vein and vasculature, as a result of radiation-induced damage. These vascular changes occurred as early as 24 h after treatment and worsened at day 2 (Figure 4). Moreover, we observed significant radiation-induced hematoma in the spinal cord white matter 2 days after a single 30 Gy irradiation, which is consistent with the literature [28]. Increase in vascular permeability occurred following irradiation, as seen by the leakage of FITC-dextran from intact vasculature. Edema and extravasation of red blood cells due to an increase in vascular permeability following irradiation has been observed previously [29], [30]. Compared with this radiation-induced vascular dysfunction, corresponding svOCT images revealed that the posterior spinal cord vein and vasculature did not suffer from significant radiation-induced structural damage over the same 2 day period. We used India ink markers placed directly on the cord surface as spatial landmarks to allow identification and serial imaging of the same vascular structures without the need for image alignment post-acquisition. These data illustrated that the SCWC could be used to follow the radiobiological response of the cord and its vasculature at morphological, microstructural, and functional levels. Fluorescence and svOCT imaging enabled clear *in vivo* longitudinal imaging of the posterior vein as well as the microscopic radial-branching vessels of approximately 25 µm diameter. svOCT was able to resolve vessels and spinal cord structure up to 500 µm in depth. Images were of high quality and had sufficient signal-to-noise ratios as determined by comparison between background fluorescence and svOCT intensities.

### Intravital Power Doppler Ultrasound, Photoacoustic and Doppler OCT Imaging of the Spinal Cord and its Vasculature

**Figure 5:**
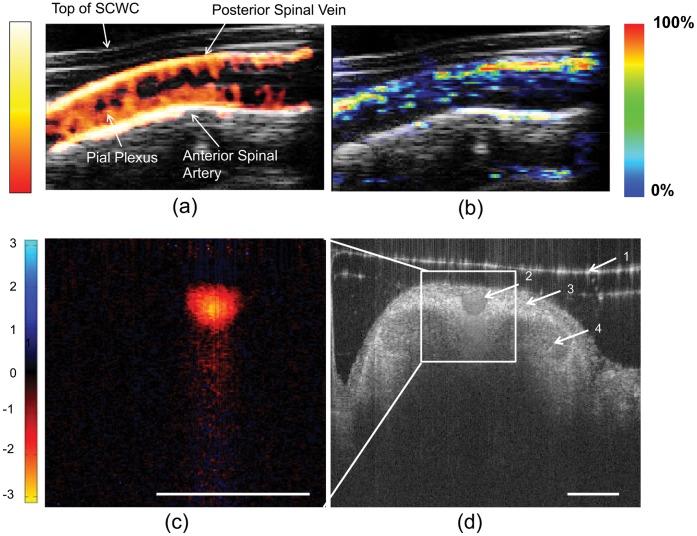
SCWC permits structural, functional and oxygenation imaging of the intact spinal cord vasculature *in situ*. (A) Power Doppler ultrasound (color) overlaid on a B-mode structural ultrasound (gray-scale) image obtained through the polycarbonate SCWC along a longitudinal section of the normal spinal cord *in vivo* (device is shown in Figure 1F). The power Doppler depicts vascular architecture in several vessels of the spinal cord. The color bar represents the signal intensity. (B) Corresponding multispectral photoacoustic imaging of the same cross section of normal spinal cord permitted *in situ* measurement of hemoglobin oxygen saturation in the anterior spinal artery and posterior spinal vein. It demonstrated that the cord is well oxygenated. The color bar represents the relative hemoglobin oxygen saturation level. (C) Cross-sectional Doppler OCT image demonstrated significant blood flow in the posterior spinal vein. The color bar represents the phase-shift of the backscattered light in radians which is proportional to the velocity of the red blood cells in the axial direction. (D) Corresponding structural OCT image of the spinal cord permitted visualization of key spinal cord features, including the glass coverslip (1), anterior spinal vein (2), white matter (3), and grey matter (4) of the intact cord. Scale Bars = 500 µm (A–D). SCWC = spinal cord window chamber. OCT = optical coherence tomography.

**Figure 6:**
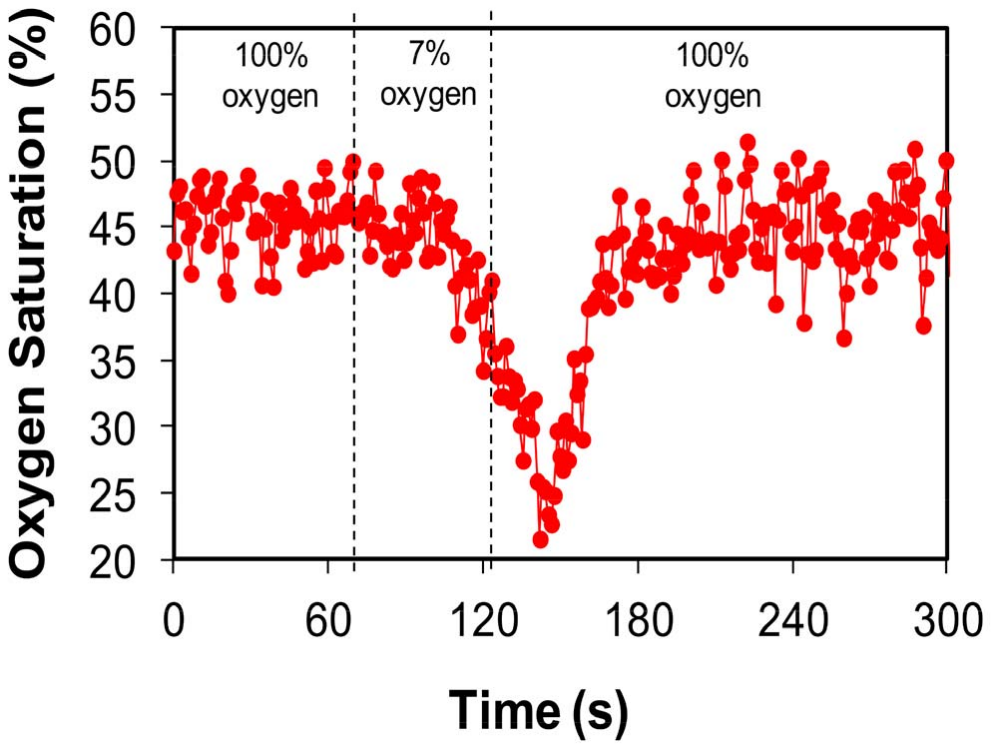
Hemoglobin oxygen saturation (sO_2_) measurement made using *in vivo* photoacoustic imaging. Baseline vascular sO_2_ was measured *in situ* for 1 minute while the animal breathed 100% oxygen mixed with 2% isoflurane. The animal was shifted to breathing 7% oxygen mixed with 2% isoflurane for an additional 1 minute. The oxygen concentration was then returned to 100%.

To further demonstrate the use of the SCWC model for other complementary imaging techniques we used power Doppler ultrasound to highlight the vascular network of the spinal cord (Figure 5A) [31]. Using the same animal, we measured sO_2_ in the intact spinal cord using multispectral photoacoustic imaging (Figure 5B). However, since power Doppler is more sensitive to the detection of small vessels compared to photoacoustics, the data shown in Figure 5A (power Doppler; *see Video S2*) displayed an increased number of vascular structures in comparison to Figure 5B (photoacoustics; *see Video S2*). Our SCWC method permitted image-based sO_2_ measurements in spinal vessels that would not have been possible without a laminectomy, since the vertebrae would have prevented effective photoacoustic imaging. Our method overcomes the impractical limitations involving the use of traditional oxygen electrodes which must be placed within the spine to measure vascular/tissue oxygenation and which only measure sO_2_ in one small tissue volume (∼1 mm^3^) at a time, requiring the needle to be moved many times for multiple measurements and possibly causing traumatic tissue damage to the cord [32]. We also demonstrated the ability to measure sO_2_ recovery in real time (Figure 6; *see Video S3*). We found that transitioning the mouse from breathing 100% to 7% oxygen for 1 minute decreased sO_2_ by approximately 23% and took approximately 30 sec to return to baseline values (Figure 6; *see Video S3*). Combining power Doppler ultrasound and spatially co-registered photoacoustic imaging of the same mouse spinal cord enabled tracking of vascular structure and sO_2_ dynamics in the same mouse over time.

We also demonstrated the feasibility of using *in vivo* Doppler OCT to image blood flow, while simultaneously capturing the cross sectional structure of the spinal cord (Figure 5C, D). Doppler OCT was able to image the posterior spinal vein only, compared with photoacoustic or power Doppler ultrasound which provided deeper tissue penetration to the anterior side of the cord. However, a major advantage of OCT imaging was the ability to spatially resolve anatomical microstructures of the spinal cord itself (Figure 5D; *see Video S4 and Video S5*), which was not possible using ultrasound imaging alone.

### SCWC Allows for Visualization of Micrometastases in the Spinal Cord

**Figure 7:**
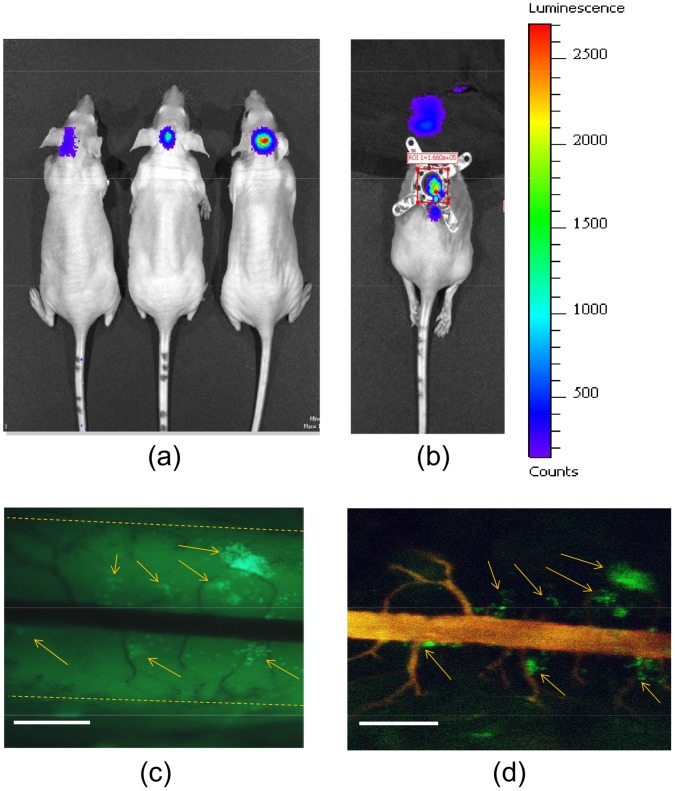
Intravital multispectral fluorescence microscopic imaging of medulloblastoma tumor metastasis to the spinal cord. (A) *In vivo* bioluminescence images of mice 7 days following intracranial tumor implantation of human WW426 medulloblastoma tumor cells, demonstrating local tumor growth. (B) SCWC was implanted 27 days after tumor implantation, when metastatic GFP+ tumor cells to the spinal cord could be seen using both BLI and intravital two-photon images (color bar indicates bioluminescence signal intensity; BLI units are photons/s/cm^2^/Sr). The head of the mouse was covered in “B” to reduce the bioluminescence signal from the brain in order to detect lower bioluminescence from the tumor micrometastases. (C) Wide-field fluorescence imaging and (D) confocal fluorescence microscopy of the SCWC-bearing mouse 28 days after initial tumor implantation (1 day post-SCWC installation). The outline of the spinal cord is highlighted with the orange dotted line in “C”. TRITC-dextran shows the posterior spinal cord vein. The arrows in “C” and “D” indicate the location of multiple tumor micrometastases in close proximity to the spinal cord vasculature. Scale bars = 1 mm. SCWC = spinal cord window chamber. BLI = bioluminescence imaging.

To further highlight an additional preclinical research use of the SCWC model, we demonstrated the intravital visualization of tumor micrometastases within the spinal cord originating from WW426 medulloblastoma cells transplanted intracranially (Figure 7A). Using bioluminescence imaging (BLI), we were able to track the migration of tumor cells down the spinal cord until they were directly under the SCWC (28 days post-transplant) (Figure 7B). Using epifluorescence microscopy, we were able to identify localized tumor micrometastases at L2–L4, immediately under the SCWC (Figure 7C). Furthermore, using TRITC-dextran to mark the spinal vessels, we observed that the metastases were in close proximity (up to 600 µm away) to the posterior spinal vein where they could access oxygen and nutrients (Figure 7D).

## Discussion

We have developed a new transparent window chamber device and surgical implantation protocol for mice that overcomes the inherent limitations of many previous *in vivo* spinal cord imaging studies. In comparison to the SCWCs developed by Farrar *et al.* and Fenrich *et al*., we have designed an alternative device, which is compatible with additional optically-enabled imaging techniques, e.g. photoacoustics, to obtain important complementary structural, functional and oxygenation information about vasculature *in vivo*. Our device design and surgical implantation methods are less complex and easily implemented for *in vivo* spinal cord imaging. Overall, this model enables direct *in vivo*, intravital multimodal imaging of healthy and diseased spinal cord and its vasculature over time. White light imaging provided high-resolution information about cord anatomy and vasculature, including hemorrhage that may occur as a result of damage cause to the cord by irradiation [28]. When combined with injectable fluorescent blood contrast agents, such as FITC- or TRITC-dextran, intravital confocal fluorescence microscopy imaging provided high intensity contrast-based images of the spinal cord vascular network for vessels as small as ∼25 µm in diameter. svOCT provided a contrast agent-free method of imaging the structure of blood vessels of the spinal cord. A limitation of both fluorescence and svOCT in imaging the spinal cord of mice is the lack of tissue-penetration to image through the full thickness of the cords, which is approximately 1.5 mm in mice [33]. Using photoacoustic imaging, the oxygenation level of the cord vasculature was quantified while power Doppler ultrasound provided visualization of vascular architecture through the complete thickness of the spinal cord. Thus, our SCWC model could be a useful tool for future imaging studies of vascular events following spinal cord injury and their contribution in pathogenesis [34].

The SCWC also permitted the use of a small animal micro-irradiator to focally treat the spinal cord directly with X–rays, followed by the imaging of the radiobiological response of the cord and the vasculature *in situ* over time. To our knowledge, this is the first attempt to study the spinal cord vascular response to radiation in mice over time using a transparent spinal cord window chamber and multimodal intravital optical imaging approach. Our results demonstrated the feasibility of this new method for studying the spinal cord vasculature and the sensitivity of this approach to radiobiological changes associated with morphology, structure and function induced by a single dose of 30 Gy. The small animal microirradiator is capable of delivering a variety of clinically-relevant radiation therapy doses and treatment regimens (e.g. single and multiple fractions) in a variety of treatment beam geometries [22]. When combined with our SCWC murine models, the microirradiator system could offer an important new preclinical experimental platform for studying the radiobiological response of the spinal cord in murine models (including in the presence of primary and metastatic tumors) longitudinally and at cellular resolution [35]. This has not been possible to date despite significant work on spinal cord radiobiology and ischemia [36], [37]. Another application of the SCWC models could be for preclinical studies of photodynamic therapy of spinal cord tumors and metastases [38], such that tumors could be irradiated by light and then imaged with optical and/or other imaging techniques (e.g. ultrasound) over time to measure the response of various tissue components. We have demonstrated that fluorescence microscopy can be used with our SCWC model to visualize tumor micrometastatic colonies in relation to the spinal cord and its vasculature. This approach could allow the study of spinal cord pathophysiology of metastatic spread as well as tumor angiogenesis at cellular-level resolution *in vivo* in future studies; however, the system will require optimization to reduce motion (breathing) artifacts during *in vivo* imaging. In addition to demonstrating the capacity to identify and track tumor cells *in vivo*, our model, when combined with high-resolution microscopy, may have the potential to observe cell-cell interactions (i.e. oligodendrocyte-neuron) and cell motility (i.e. leukocyte trafficking through diapedesis), which may be beneficial for visualizing CNS regeneration or monitoring a localized inflammatory responses, respectively. There is also a growing body of evidence pointing to the existence of a subset of tumor cells with high tumorigenic potential in many spine cancers that exhibit characteristics similar to stem cells [39]. Our intravital SCWC experimental model could be useful for such emerging biological studies.

While there have been previous studies reported on *in vivo* optical imaging of the spinal cord, they have required repeated surgeries to remove the skin to access the cord for longitudinal imaging [10], [11], [12], [13], [14], [15], [16], [40], [41]. Recently, two studies have demonstrated the implementation of a transparent window chamber approach to facilitate serial optical imaging of the spinal cord *in vivo*; however, their SCWC have differed in materials and design in comparison to our model [16], [17]. In contradistinction to our design, their spinal chamber incorporated metal components situated deep in the vertebral column, and were located adjacent to the vertebrae (although not in direct contact with the spinal cord). These metal components stabilized the cordduring imaging and the authors state that this technique produced a moderate inflammatory response in the cord (microglial density was increased at both 1d and 7d post-implantation). In our model, we developed a chamber that was not fixed to the vertebrae, yet still permitted optical imaging with few motion artifacts. Our design involved components that are located superficially to the spinal cord (implanted and stablizied in the dorsal skin/muscle), preventing any contact between the spinal cord and the SCWC device. In addition, unlike the Farrar and Fenrich designs, which include metallic components, our plastic spinal chamber enabled the use of real-time multispectral photoacoustic and ultrasound imaging in addition to the fluoerscence and svOCT imaging *in vivo*. This is the first time that OCT imaging has been performed longitudinally in the *in vivo* spinal cord, and the first time photoacoustic imaging has ever been used to image the spinal cord *in vivo*. Thus, using a plastic chamber increased the number of additional compatible imaging techniques that could be used to study the spinal cord in greater detail. The transparent SCWC model could offer a method of precise delivery of either ionizing or ablative optical energy to the cord with a potential role in studies of cellular-based regenerative enhancement of spinal cord injury repair [42].

Our SCWC experimental model is practical, easy to use, and involves a single surgical procedure to implant the window chamber, which minimizes the possibility of focal traumatic damage caused by repeated surgical exposure of the spinal cord. In contrast to Farrar *et al.*, we did not observe considerable local tissue inflammation in the cord, as demonstrated by negligible changes in Iba-1 expression in cord tissues after implantation (Figure 2). This suggests that a SCWC implanted more superficial/dorsal, rather than directly adjacent to the vertebral column, may be a more effective model that does not induce significant inflammation. Further, no motor-function deficits or neuropathology were observed in the chamber-bearing mice (S*ee Video S1*). Importantly, the metal and plastic spinal chambers were designed to be lightweight and to minimize discomfort to the animal as well as interference to the normal activities (e.g. feeding, drinking, grooming, locomotion, etc.) of the animal. Therefore, the SCWC we have developed for long term chronic imaging of the normal and diseased spinal cord, including after radiation treatment, is a robust, reproducible, and a useful murine experimental model for imaging the spinal cord and its vasculature with optically-enabled intravital imaging techniques.

Additionally, we have recently extended the SCWC model from mice to rats (*See File S1 and Figure S1*). By developing a rat SCWC model, we open the possibility of experimental studies in a larger murine model that more accurately represents the human spinal cord. This is important for studying the pathophysiology and development of the cystic cavity following spinal cord injury (*See File S1*) [43], [44].

While our SCWC model overcomes previous challenges in optical imaging of the living spinal cord, we have recognized a few limitations. Firstly, our chambers were designed with an inner diameter of 8 mm, which is wide enough to fit standard microscope objective lens (from 1X –60X), yet had an overall size and profile that were small enough to avoid restricting animal movement after implantation. However, an 8 mm diameter transparent window to the spinal cord allows microscopic observation of the spinal cord for only 2 to 3 spinal segments at the most. Increasing the size of the chamber would require more vertebral segments to be removed and we found this risked fracture of the vertebral column. For the purposes of longitudinal and localized optical imaging of tissue, cellular and vascular changes to the cord following traumatic and/or localized damage (i.e. spinal cord injury, stroke, spinal tumors), an 8 mm diameter window is sufficient. However, for the study of neurological diseases, such as multiple sclerosis or amyotrophic lateral sclerosis, which have widespread effects along the spinal cord, *in vivo* optical imaging using our SCWC setup may not be appropriate to assess the global deterioration of the whole cord [45], [46]. Secondly, while intravital confocal fluorescence microscopy (with exogenous blood contrast agents) and svOCT (without contrast agents) provide high-resolution imaging of the microvasculature which cannot be achieved by other imaging techniques such as microCT or MRI, these optical imaging methods cannot image through the full thickness of the spinal cord. Therefore, only pial and white matter vessels are accessible using fluorescence and svOCT, while the majority of vascular structures in the grey matter remain a challenge for optical imaging. One possible alternative is the use of photoacoustic imaging which uses high-frequency ultrasound to image through the entire spinal cord following pulsed laser excitation, including vessels of the grey matter. While yielding useful structural information about tissue and vasculature, intravital multispectral photoacoustic imaging also provides important functional information of spinal cord vascular oxygenation status non-invasively over time.

Nevertheless, our research – in conjunction with previous reports by Farrar *et al*. and Fenrich *et al*. – substantiate the need and confirm the technical feasibility for such unique murine window chamber models for *in vivo* longitudinal multimodal imaging of the spinal cord. Future preclinical studies of the healthy, diseased or injured spinal cord will benefit from the availability of such robust experimental animal models and their ability to exploit powerful multimodal and intravital imaging techniques.

## Supporting Information

Figure S1 Spinal cord window chamber designed for imaging of the rat cord and vasculature. (A) The spinal cord window chamber device was designed and printed in polycarbonate with a metal ring to secure the 12-mm coverglass slip. (B) The SCWC device, shown from a different angle, has lateral arms which retract the dorsal muscles of the vertebrae and keep the spinal cord exposed over days-to-weeks- for longitudinal optical imaging. (C) The SCWC device is surgically implanted over the exposed spinal cord providing a window for direct spinal cord imaging, following a two-level laminectomy at T6–T7. (D) Images of the rat spinal cord were taken 3 days after SCWC implantation. Intravital white light at 2X magnification (left panel), and 6X magnification inset of intravenous FITC-dextran (right panel) images are shown. Scale bars = 1 cm. SCWC = spinal cord window chamber.
(TIF)

File S1 (DOCX)

Video S1 Behavioural and functional observation of mice 28 day post-SCWC implantation. Athymic nude mice had spinal cord window chambers implanted and were followed for 1 month to examine their behaviour, motor function, grooming, and eating habits, as well as to document any necrosis, inflammation or infection surrounding the implantation site. No motor/behavioural deficits were observed in the 28 day period. Similarly, no observable inflammation, necrosis or infection resulted from spinal cord window chamber (SCWC) implantation.
(MP4)

Video S2 Photoacoustic and Power Doppler imaging. Co-registered power Doppler and oxygen saturation (sO_2_) measurements of the spinal cord. Three-dimensional power Doppler image of the spinal cord vasculature shown in orange demonstrates the ability to image multiple vascular structures within the spinal cord. Longitudinal section of the cord is illustrated by the structural ultrasound image and overlaid photoacoustic image. Color bar indicates the relative sO_2_ level of the vasculature. Imaging was performed while the mouse was breathing 100% oxygen mixed with 2% isoflurane.
(WMV)

Video S3 Spinal cord O_2_ saturation monitoring by photoacoustic imaging. Two-dimensional cross-section of the spinal cord within the window chamber. Ultrasound structural image (left) shows the outline of the window chamber as well as the artificial dura that cover the spinal cord. The rectangle indicates the region where photoacoustic image was acquired, and the circular region of interest indicates the area that photoacoustic signal intensity was measured. Photoacoustic image (right) displays the spinal cord vasculature. Color bar indicates the relative oxygenation level of the vasculature, and the scale bar illustrates the depth of imaging from the transducer head. The animal’s anaesthetic mixture was shifted from 100% to 7% oxygen for 1 minute, which corresponds to the frame 58 to 103 (out of total 248 frames acquired) in this video.
(AVI)

Video S4 3D OCT. Reconstructed three-dimensional structural OCT image acquired over 2.5 mm×3 mm regions of the cord within the window chamber. Arterial spinal vein and the spinal cord structure can be seen throughout the region of interest. OCT = optical coherence tomography.
(MPG)

Video S5 3D OCT. The same reconstructed three-dimensional structural OCT image as in Video S4 was made transparent for better visualization to highlight the structure of the spinal cord. OCT = optical coherence tomography.
(MPG)

## References

[1] Stroman PW (2005) Magnetic Resonance Imaging of Neuronal Function in the Spinal Cord: Spinal fMRI. Clinical Medicine & Research 3: 146–156. 16160069 10.3121/cmr.3.3.146 PMC1237156

[2] Braun IF, Raghavendra BN, Kricheff II (1983) Spinal cord imaging using real-time high-resolution ultrasound. Radiology 147: 459–465. 6340159 10.1148/radiology.147.2.6340159

[3] Moseley ME, Cohen Y, Kucharczyk J, Mintorovitch J, Asgari HS, et al (1990) Diffusion-weighted MR imaging of anisotropic water diffusion in cat central nervous system. Radiology 176: 439–445. 2367658 10.1148/radiology.176.2.2367658

[4] McAfee PC, Bohlman HH, Han JS, Salvagno RT (1986) Comparison of Nuclear Magnetic Resonance Imaging and Computed Tomography in the Diagnosis of Upper Cervical Spinal Cord Compression. Spine 11: 295–304. 3750059 10.1097/00007632-198605000-00001

[5] Tench CR, Morgan PS, Jaspan T, Auer DP, Constantinescu CS (2005) Spinal Cord Imaging in Multiple Sclerosis. Journal of Neuroimaging 15: 94S–102S. 16385022 10.1177/1051228405283292

[6] Madi S, Flanders AE, Vinitski S, Herbison GJ, Nissanov J (2001) Functional MR Imaging of the Human Cervical Spinal Cord. American Journal of Neuroradiology 22: 1768–1774. 11673177 PMC7974439

[7] Misgeld T, Kerschensteiner M (2006) In vivo imaging of the diseased nervous system. Nat Rev Neurosci 7: 449–463. 16715054 10.1038/nrn1905

[8] Dommisse GF (1974) The Blood Supply of the Spinal Cord: A Critical Vascular Zone in Spinal Surgery. J Bone Joint Surg Br 56-B: 225–235. 4854669

[9] Marcus M, Heistad D, Ehrhardt J, Abboud F (1977) Regulation of total and regional spinal cord blood flow. Circulation Research 41: 128–134. 862136 10.1161/01.res.41.1.128

[10] Kerschensteiner M, Schwab ME, Lichtman JW, Misgeld T (2005) In vivo imaging of axonal degeneration and regeneration in the injured spinal cord. Nat Med 11: 572–577. 15821747 10.1038/nm1229

[11] Davalos D, Lee JK, Smith WB, Brinkman B, Ellisman MH, et al (2008) Stable in vivo imaging of densely populated glia, axons and blood vessels in the mouse spinal cord using two-photon microscopy. Journal of Neuroscience Methods 169: 1–7. 18192022 10.1016/j.jneumeth.2007.11.011 PMC2647134

[12] Johannssen HC, Helmchen F (2010) In vivo Ca2+ imaging of dorsal horn neuronal populations in mouse spinal cord. The Journal of Physiology 588: 3397–3402. 20660563 10.1113/jphysiol.2010.191833 PMC2988506

[13] Kim JV, Jiang N, Tadokoro CE, Liu L, Ransohoff RM, et al (2010) Two-photon laser scanning microscopy imaging of intact spinal cord and cerebral cortex reveals requirement for CXCR6 and neuroinflammation in immune cell infiltration of cortical injury sites. Journal of Immunological Methods 352: 89–100. 19800886 10.1016/j.jim.2009.09.007 PMC2808463

[14] Dray C, Rougon Gv, Debarbieux F (2009) Quantitative analysis by in vivo imaging of the dynamics of vascular and axonal networks in injured mouse spinal cord. Proceedings of the National Academy of Sciences 106: 9459–9464. 10.1073/pnas.0900222106 PMC2685250 19470644

[15] Cadotte DW, Mariampillai A, Cadotte A, Lee KKC, Kiehl T-R, et al (2012) Speckle variance optical coherence tomography of the rodent spinal cord: in vivo feasibility. Biomed Opt Express 3: 911–919. 22567584 10.1364/BOE.3.000911 PMC3342196

[16] Farrar MJ, Bernstein IM, Schlafer DH, Cleland TA, Fetcho JR, et al.. (2012) Chronic in vivo imaging in the mouse spinal cord using an implanted chamber. Nature Methods advance online publication. 10.1038/nmeth.1856 PMC3429123 22266542

[17] Fenrich KK, Weber P, Hocine M, Zalc M, Rougon G, et al.. (2012) Long-term in vivo imaging of normal and pathological mouse spinal cord with sub-cellular resolution using implanted glass windows. The Journal of Physiology. 10.1113/jphysiol.2012.230532 PMC3476626 22641787

[18] Xu M, Wang LV (2006) Photoacoustic imaging in biomedicine. Review of Scientific Instruments 77: 041101–041122.

[19] Wang X, Xie X, Ku G, Wang LV, Stoica G (2006) Noninvasive imaging of hemoglobin concentration and oxygenation in the rat brain using high-resolution photoacoustic tomography. Journal of Biomedical Optics 11: 024015–024019. 16674205 10.1117/1.2192804

[20] Ntziachristos V (2010) Going deeper than microscopy: the optical imaging frontier in biology. Nat Meth 7: 603–614. 10.1038/nmeth.1483 20676081

[21] Shtoyerman E, Arieli A, Slovin H, Vanzetta I, Grinvald A (2000) Long-Term Optical Imaging and Spectroscopy Reveal Mechanisms Underlying the Intrinsic Signal and Stability of Cortical Maps in V1 of Behaving Monkeys. The Journal of Neuroscience 20: 8111–8121. 11050133 10.1523/JNEUROSCI.20-21-08111.2000 PMC6772749

[22] Clarkson R, Lindsay PE, Ansell S, Wilson G, Jelveh S, et al (2011) Characterization of image quality and image-guidance performance of a preclinical microirradiator. Medical Physics 38: 845–856. 21452722 10.1118/1.3533947 PMC3188651

[23] Imai Y, Ibata I, Ito D, Ohsawa K, Kohsaka S (1996) A Novel Geneiba1in the Major Histocompatibility Complex Class III Region Encoding an EF Hand Protein Expressed in a Monocytic Lineage. Biochemical and Biophysical Research Communications 224: 855–862. 8713135 10.1006/bbrc.1996.1112

[24] Sasaki Y, Ohsawa K, Kanazawa H, Kohsaka S, Imai Y (2001) Iba1 Is an Actin-Cross-Linking Protein in Macrophages/Microglia. Biochemical and Biophysical Research Communications 286: 292–297. 11500035 10.1006/bbrc.2001.5388

[25] Yu W, Fehlings M (2011) Fas/FasL-mediated apoptosis and inflammation are key features of acute human spinal cord injury: implications for translational, clinical application. Acta Neuropathologica 122: 747–761. 22038545 10.1007/s00401-011-0882-3 PMC3224722

[26] Popovich PG, Wei P, Stokes BT (1997) Cellular inflammatory response after spinal cord injury in sprague-dawley and lewis rats. The Journal of Comparative Neurology 377: 443–464. 8989657 10.1002/(sici)1096-9861(19970120)377:3<443::aid-cne10>3.0.co;2-s

[27] Donnelly DJ, Popovich PG (2008) Inflammation and its role in neuroprotection, axonal regeneration and functional recovery after spinal cord injury. Experimental Neurology 209: 378–388. 17662717 10.1016/j.expneurol.2007.06.009 PMC2692462

[28] Powers BE, Beck ER, Gillette EL, Gould DH, LeCouter RA (1992) Pathology of radiation injury to the canine spinal cord. International Journal of Radiation Oncology*Biology*Physics 23: 539–549. 10.1016/0360-3016(92)90009-7 1612954

[29] Hornsey S, Myers R, Jenkinson T (1990) The reduction of radiation damage to the spinal cord by post-irradiation administration of vasoactive drugs. International Journal of Radiation Oncology*Biology*Physics 18: 1437–1442. 10.1016/0360-3016(90)90319-f 2370194

[30] Siegal T, Pfeffer MR (1995) Radiation-induced changes in the profile of spinal cord serotonin, prostaglandin synthesis, and vascular permeability. International Journal of Radiation Oncology*Biology*Physics 31: 57–64. 10.1016/0360-3016(94)E0305-4 7527800

[31] Bilgen M, Al-Hafez B (2006) Comparison of spinal vasculature in mouse and rat: investigations using MR angiography. Neuroanatomy 5: 12–16.

[32] Nix W, Capra N, Erdmann W, Halsey J (1976) Comparison of vascular reactivity in spinal cord and brain. Stroke 7: 560–563. 1006728 10.1161/01.str.7.6.560

[33] Anderson CR, Ashwell KWS, Collewijn H, Conta A, Harvey A, et al.. (2009) The Spinal Cord: A Christopher and Dana Reeve Foundation Text and Atlas; Charles W, George P, Gulgun KayaliogluA2 - Charles Watson GP, Gulgun K, editors. San Diego: Academic Press. v p.

[34] Sinescu C, Popa F, Grigorean V, Onose G, Sandu A, et al. (2010 ) Molecular basis of vascular events following spinal cord injury. Journal of Medicine and Life 3: 254–261. 20945816 PMC3018992

[35] Sahgal A, Bilsky M, Chang EL, Ma L, Yamada Y, et al (2011) Stereotactic body radiotherapy for spinal metastases: current status, with a focus on its application in the postoperative patient. Journal of Neurosurgery: Spine 14: 151–166. 21184635 10.3171/2010.9.SPINE091005

[36] Kirkpatrick JP, van der Kogel AJ, Schultheiss TE (2010) Radiation Dose-Volume Effects in the Spinal Cord. International Journal of Radiation Oncology*Biology*Physics 76: S42–S49. 10.1016/j.ijrobp.2009.04.095 20171517

[37] van der Kogel AJ (1993) Dose-volume effects in the spinal cord. Radiotherapy and Oncology 29: 105–109. 8310135 10.1016/0167-8140(93)90234-y

[38] Liu TW, Akens MK, Chen J, Wise-Milestone L, Wilson BC, et al (2011) Imaging of Specific Activation of Photodynamic Molecular Beacons in Breast Cancer Vertebral Metastases. Bioconjugate Chemistry 22: 1021–1030. 21585206 10.1021/bc200169x

[39] Hsu W, Mohyeldin A, Shah SR, Gokaslan ZL, Quinones-Hinojosa A (2012) Role of Cancer Stem Cells in Spine Tumors: Review of Current Literature. Neurosurgery. E-pub ahead of print. doi:10.1227/NEU.1220b1013e3182532e3182571. 10.1227/NEU.0b013e3182532e71 22418583

[40] Bhavna Y, Ertürk A, Hellal F, Nadrigny F, Hurtado A, et al (2009) Chronically CNS-Injured Adult Sensory Neurons Gain Regenerative Competence upon a Lesion of Their Peripheral Axon. Current Biology 19: 930–936. 19409789 10.1016/j.cub.2009.04.017

[41] Dibaj P, Nadrigny F, Steffens H, Scheller A, Hirrlinger J, et al (2010) NO mediates microglial response to acute spinal cord injury under ATP control in vivo. Glia 58: 1133–1144. 20468054 10.1002/glia.20993

[42] Fehlings M, Vawda R (2011) Cellular Treatments for Spinal Cord Injury: The Time is Right for Clinical Trials. Neurotherapeutics 8: 704–720. 22002087 10.1007/s13311-011-0076-7 PMC3210356

[43] Thuret S, Moon LDF, Gage FH (2006) Therapeutic interventions after spinal cord injury. Nat Rev Neurosci 7: 628–643. 16858391 10.1038/nrn1955

[44] Byrnes KR, Fricke ST, Faden AI (2010) Neuropathological differences between rats and mice after spinal cord injury. Journal of Magnetic Resonance Imaging 32: 836–846. 20882614 10.1002/jmri.22323 PMC2949295

[45] Lassmann H (2005) Multiple Sclerosis Pathology: Evolution of Pathogenetic Concepts. Brain Pathology 15: 217–222. 16196388 10.1111/j.1750-3639.2005.tb00523.x PMC8095927

[46] Rowland LP, Shneider NA (2001) Amyotrophic Lateral Sclerosis. New England Journal of Medicine 344: 1688–1700. 11386269 10.1056/NEJM200105313442207

