# Supplementary figures and images for: A Spinal Cord Window Chamber Model for In Vivo Longitudinal Multimodal Optical and Acoustic Imaging in a Murine Model
Source: PLoS One. 2013 Mar 14;8(3):e58081. doi: 10.1371/journal.pone.0058081 (PMC3597636; doi:10.1371/journal.pone.0058081)

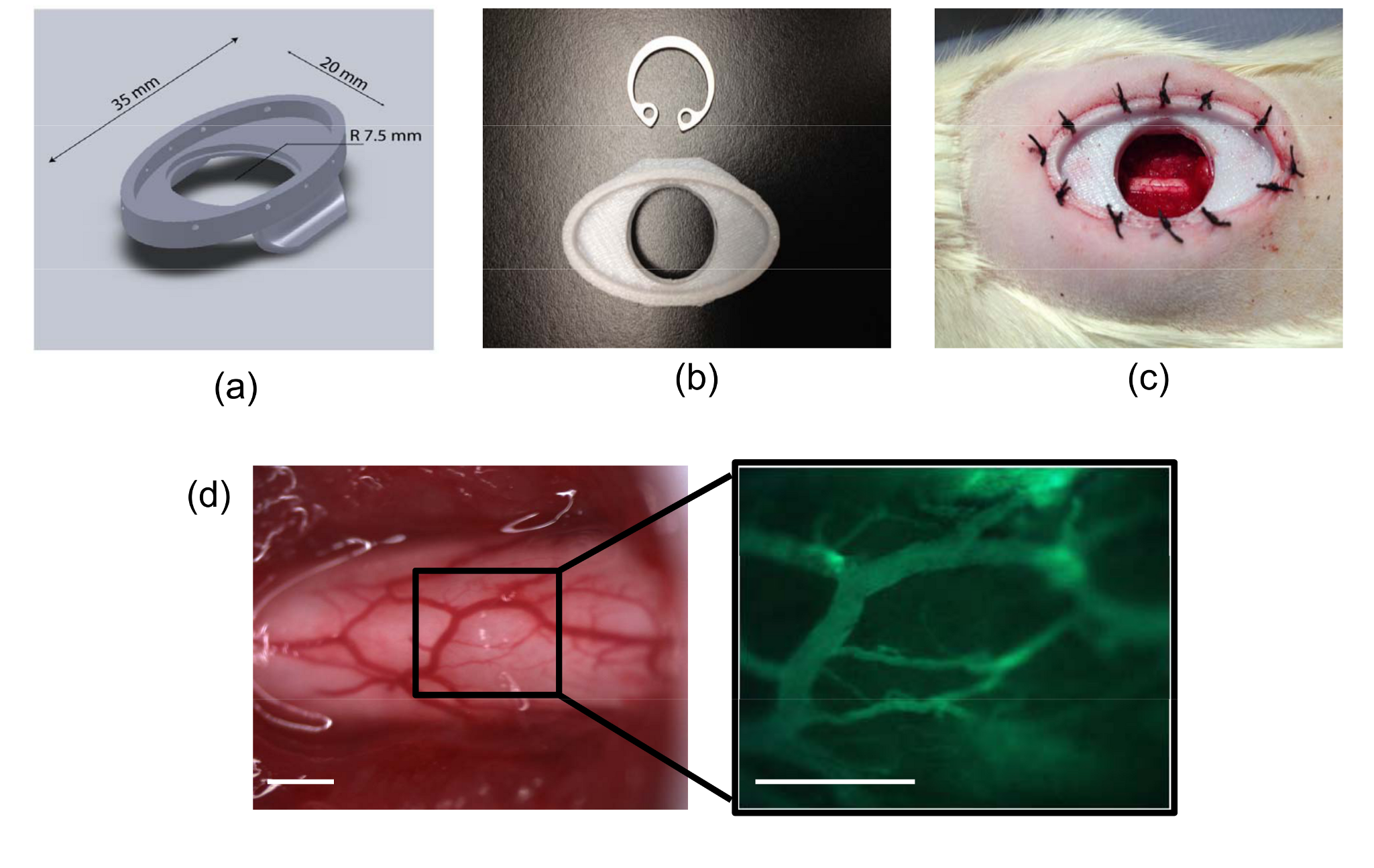

Supplement: Figure S1 — Spinal cord window chamber designed for imaging of the rat cord and vasculature. (A) The spinal cord window chamber device was designed and printed in polycarbonate with a metal ring to secure the 12-mm coverglass slip. (B) The SCWC device, shown from a different angle, has lateral arms which retract the dorsal muscles of the vertebrae and keep the spinal cord exposed over days-to-weeks- for longitudinal optical imaging. (C) The SCWC device is surgically implanted over the exposed spinal cord providing a window for direct spinal cord imaging, following a two-level laminectomy at T6–T7. (D) Images of the rat spinal cord were taken 3 days after SCWC implantation. Intravital white light at 2X magnification (left panel), and 6X magnification inset of intravenous FITC-dextran (right panel) images are shown. Scale bars = 1 cm. SCWC = spinal cord window chamber. (TIF) [file pone.0058081.s001.tif]
