# Supplementary material for: A Spinal Cord Window Chamber Model for In Vivo Longitudinal Multimodal Optical and Acoustic Imaging in a Murine Model
Source: PLoS One. 2013 Mar 14;8(3):e58081. doi: 10.1371/journal.pone.0058081 (PMC3597636; doi:10.1371/journal.pone.0058081)
Supplement: File S1 — (DOCX) [file pone.0058081.s002.docx]

**A spinal cord window chamber model for *in vivo* longitudinal multimodal optical and acoustic imaging in a murine model**

Sarah A. Figley ^1,2^**^†^** & Yonghong Chen ^3^**^†^** , Azusa Maeda ^4^, Leigh Conroy ^4^, Jesse D. McMullen ^3^, Jason I. Silver ^3^, Shawn Stapleton ^4^_,_ Alex Vitkin ^3,4,5^, Patricia Lindsay ^5^, Kelly Burrell ^2^, Gelareh Zadeh ^2^, Michael G. Fehlings ^1,2^, and Ralph S. DaCosta ^3,4,5^*

† Equal contribution.

^1^ *Institute of Medical Science, University of Toronto, Toronto, Ontario, Canada.*

*^2^ Toronto Western Research Institute, Krembil Neuroscience Program, University Health Network, Toronto, Ontario, Canada.*

*^3^ Ontario Cancer Institute, University Health Network, Princess Margaret Hospital, Toronto, Ontario, Canada.*

*^4^ Department of Medical Biophysics, University of Toronto, Toronto, Ontario, Canada.*

*^5^ Department of Radiation Physics, University Health Network, Princess Margaret Hospital, Toronto, Ontario, Canada.*

**Corresponding author:*

Dr. Ralph S. DaCosta

Cancer Care Ontario Research Chair in Cancer Imaging;

Ontario Cancer Institute, University Health Network;

Department of Medical Biophysics,

Faculty of Medicine, University of Toronto;

Princess Margaret Hospital,

610 University Avenue, Rm 7-416,

Toronto, Ontario, Canada M5G 2M9,

Tel: (416) 581-8645

Fax: (416)-946-6529

Email: *rdacosta@uhnres.utoronto.ca*

**SUPPORTING INFORMATION – METHODS:**

*Rat Spinal Cord Window Chamber Design and Installation*: For rats, we designed a slightly oval-shaped ABS-polycarbonate SCWC device with curved lateral arms which retract the dorsal muscles of the vertebrae and keep the spinal cord (Figure S1A, B). The rat spinal chambers had a 12-mm window diameter, diameter coverglass and metal ring clamp, with the implant weighing 1.90 g.

Adult female Wistar rats (Charles River, Montreal, Canada) each weighing between 250-300 g were deeply anesthetized using 4% isoflurane, and were sedated for the remainder of the surgery with 2% isoflurane. A 5-6 mm incision of the skin was made along the dorsal midline to expose the dorsal muscles, and the muscle layers were opened and retracted separately to expose the vertebral column. Animals received a two-level laminectomy of mid-thoracic vertebral segments T6-T7. A rat SCWC device was installed directly over the exposed spinal cord. The rat spinal cord chamber has been designed with lateral arms to retract the muscle layers and keep the exposed cord clear. Rats were imaged 3 days after installation of the SCWC device using white light and fluorescent microscopy. Animals were housed individually for up to 5 days and were allowed access to food and water *ad libitum*.

**SUPPORTING INFORMATION – RESULTS AND DISCUSSION:**

As an alternative to the mouse SCWC model, we also developed a similar SCWC device for use in rats, which have spinal cords that are approximately twice the diameter/thickness of a mouse spinal cord [[30](#_ENREF_30)]. In our preliminary experiments, we have demonstrated the feasibility of using a SCWC constructed from polycarbonate material for minimally-invasive longitudinal imaging of the rat spinal cord and its vasculature. Rats implanted with the spinal chambers survived for up to 7 days after implantation without behavioral problems, locomotor function deficits, or infection. Window chambers remained optically clear for the duration of the experiments, and new coverslips were easily installed before and/or after imaging sessions. Figure S1D shows white light and corresponding high-resolution vascular images obtained intravitally using FITC-dextran injected intravenously prior to fluorescence imaging at 3 days following SCWC implantation.

While the SCWC model was directly transferrable to white light and fluorescent imaging in the rat model, we encountered a limitation in the rat that prevented acquisition of speckle variance OCT and photoacoustic images from the same site on the cord. This was due to significant motion of the spinal cord caused by a combined effect of breathing and beating of the heart during *in vivo* imaging. Both imaging modalities – svOCT and photoacoustics – are highly sensitive to tissue motion artifacts; however, a recent publication by Cadotte *et al.* illustrates the optimized svOCT imaging in the rat spinal cord, and reports a significant reduction of spinal cord motion artifacts based on newer svOCT imaging instrumentation [[15](#_ENREF_15)]. Future studies that combine the SCWC model with svOCT and/or photoacoustic imaging may provide new ways to study the normal *in vivo* rat spinal cord vasculature, or changes in the vasculature following disease onset or traumatic injury.
